# Cost of treatment for breast cancer in central Vietnam

**DOI:** 10.3402/gha.v6i0.18872

**Published:** 2013-02-04

**Authors:** Nguyen Hoang Lan, Wongsa Laohasiriwong, John Frederick Stewart, Nguyen Dinh Tung, Peter C. Coyte

**Affiliations:** 1 Graduate School, Khon Kaen University, Khon Kaen, Thailand; 2 Hue College of Medicine and Pharmacy, Hue University, Hue city, Vietnam; 3 Faculty of Public Health and Board Committee of Research and Training Center for Enhancing Quality of Life of Working Age People (REQW), Khon Kaen University, Khon Kaen, Thailand; 4 Department of Economics, University of North Carolina, Chapel Hill, USA; 5 Department of Oncology, Hue Central Hospital, Hue city, Vietnam; 6 Institute of Health Policy, Management and Evaluation, University of Toronto, Toronto, Ontario, Canada

**Keywords:** breast cancer, direct medical cost, health care payer, Vietnam

## Abstract

**Background:**

In recent years, cases of breast cancer have been on the rise in Vietnam. To date, there has been no study on the financial burden of the disease. This study estimates the direct medical cost of a 5-year treatment course for women with primary breast cancer in central Vietnam.

**Methods:**

Retrospective patient-level data from medical records at the Hue Central Hospital between 2001 and 2006 were analyzed. Cost analysis was conducted from the health care payers’ perspective. Various direct medical cost categories were computed for a 5-year treatment course for patients with breast cancer. Costs, in US dollars, discounted at a 3% rate, were converted to 2010 after adjusting for inflation. For each cost category, the mean, standard deviation, median, and cost range were estimated. Median regression was used to investigate the relationship between costs and the stage, age at diagnosis, and the health insurance coverage of the patients.

**Results:**

The total direct medical cost for a 5-year treatment course for breast cancer in central Vietnam was estimated at $975 per patient (range: $11.7–$3,955). The initial treatment cost, particularly the cost of chemotherapy, was found to account for the greatest proportion of total costs (64.9%). Among the patient characteristics studied, stage at diagnosis was significantly associated with total treatment costs. Patients at later stages of breast cancer did not differ significantly in their total costs from those at earlier stages however, but their survival time was much shorter. The absence of health insurance was the main factor limiting service uptake.

**Conclusion:**

From the health care payers’ perspective, the Government subsidization of public hospital charges lowered the direct medical costs of a 5-year treatment course for primary breast cancer in central Vietnam. However, the long treatment course was significantly influenced by out-of-pocket payments for patients without health insurance.

Breast cancer is the most common cancer among women worldwide (1). Advances in screening programs and treatment methods have improved the life expectancy of patients with breast cancer (2). From a societal perspective, the economic burden of this disease has been reported in several studies using available data in developed countries; however, the direct medical cost is thought to make the smallest contribution to total costs, accounting for 50% of indirect costs (morbidity and mortality) (3, 4). These medical costs, while a small proportion of overall costs, may overwhelm patients, particularly those with lower incomes. A study by Chu et al. in Taiwan found that, among major cancers, breast cancer was associated with the largest total lifetime medical costs at 5,046 million TWD (5). In the United States, Barron et al. (2008), using pooled administrative data for five US health plans, estimated costs of breast cancer treatment per patient per month at $2,896 or approximately at $34,752 per year (6). Medical costs were found to increase with the stage of the disease (3, 4, 6, 7). In 1996, Legorreta et al., using US medical records and claims data, determined that costs over a 4-year period for patients with stage III breast cancer averaged more than $60,000, whereas costs were lower in patients at stage 0, I, and IV at $19,000, $21,000, and approximately $40,000, respectively (8).

**Policy recommendations from the study**

- Earlier diagnosis of breast cancer should be enabled through screening programs to increase treatment effectiveness and to save health care resources.
- Universal health insurance coverage should be given more attention, especially since public hospital charges are expected to increase in the near future.
- The Vietnamese government should have a policy to support cancer patients when the cost of their illness is expected to exceed their ability to pay (with or without health insurance).

Breast cancer has also become an important public health problem in Vietnam. The incidence rate increased from 13.8 per 100,000 women in 2000 to 28.1 per 100,000 women in 2010. In 2010, it was reported that there were 12,533 women with breast cancer in the country (9). In Vietnam, as in many other developing countries, breast cancer was characterized by late presentation, young patients, and low survival rates (10–13). Recent studies in Vietnam have revealed that poor knowledge and awareness among the general public is a major contributor to those problems (13). However, the financial burden of treatment of breast cancer has not yet been considered as a contributing factor. The objective of this study is to provide estimates of the total direct medical costs for breast cancer treatment in central Vietnam. The findings can contribute to models of cost-effectiveness analysis of interventions for breast cancer and can support policy adaptations for better care of the women with this disease in Vietnam.

## Methods

### Study design

A retrospective study was designed to estimate the cost of treatment for women with breast cancer in central Vietnam. Medical records of patients with a code of C 50 (ICD-10 version) admitted to Hue Central Hospital (HCH) between 2001 and 2006 were searched to identify breast cancer patients presenting in those years (14). Data, from medical records and participant's recall, on the patients’ costs for medical care for breast cancer were collected for a period of 5 years following primary diagnosis. Calculation of expenditure for breast cancer treatment was based on actual patient-level cost data, excluding the costs for herbal treatment or unpaid family care, because it is difficult to control these costs, especially in the context of the many variations of herbal medicines in Vietnam. Unit costs during the period of study were provided from the financial department of the hospital. The direct medical cost of treatment for women with breast cancer was analyzed from the perspective of health care payers, including the cost borne by patients and health insurance providers. The payment amount or hospital fee included the cost of medications and materials used in clinical practice together with the user fees borne by patients. User fees are based on a decree on partial collection of public hospital fees as regulated by the Vietnamese government (1994) and the decree's revisions (15, 16). The direct non-medical costs (e.g. travel, accommodation, time) and indirect costs (e.g. lost income or premature death due to the disease) were not included in these calculations.

### Data sources

The data were collected from two sources:

Primary data: Patients or their relatives (if patients were deceased) were interviewed directly using a structured questionnaire. Data on sociodemographic characteristics, the type of initial treatment received during hospitalization as well as during a 5-year follow-up period after the initial treatment, and compliance with the treatment regime for follow-up care were collected. For deceased patients, the date and cause of death were also noted.

Secondary data: The paper charts stored at Hue Central Hospital of patients with breast cancer were examined to obtain personal information (e.g. name, age, home address), date of admission, diagnosis and stage, treatment regimes, itemized invoices, and health insurance participation. Unit costs for treatments received over the study period were acquired from the hospital's finance department.

### Study population

HCH was selected as the site for the study. This hospital is located in the city of Hue, the capital of the central coastal Thua Thien Hue province. HCH is one of the three largest hospitals under the management of the Ministry of Health in Vietnam. The hospital is a national general hospital and a leading referral hospital in the central region. The medical records of patients presenting with breast cancer at HCH were screened to identify those meeting the following criteria – inpatient admission to the hospital between January 2001 and January 2006, residents of Thua Thien Hue province, diagnosis of primary breast cancer identified in the paper charts by the occurrence of code C 50 (ICD-10 version), and evidence of stage of breast cancer according to the tumor/nodes/metastasis (TNM) staging system of the Union for International Cancer Control (UICC) (17); 160 patients were identified according to the criteria for inclusion and tracked until December 31, 2010, to determine their 5-year survival time. The time period from 2001 to 2006 was used to obtain a more comprehensive sample of patients at various stages of breast cancer from different age groups. This long time period was necessary because the incidence rate of the disease in Thua Thien Hue province was not high (9). The results of follow-up left 129 eligible patients for whom costs could be analyzed. The main reason for 31 cases being lost to follow-up was migration to another treatment site.

### Diagnosis to define breast cancer

In HCH, between 2001 and 2006, major laboratory tests were often requested to define breast cancer, including breast ultrasound, hematogram, CA 15.3, tumor biopsy, and cytological tests. Some patients might also have had mammography and estrogen receptor (ER) tests, progesterone receptor (PR) tests, and Her 2-Neu tests from other health facilities in the province or in the country (personal communication, Dr. Nguyen Dinh Tung, Oncology Department, Hue Central Hospital). In this study, only those tests recorded in the patients’ medical records were included.

### Treatment pattern for breast cancer between 2001 and 2006

During the study period, advanced medical equipment and new medications were in limited use in public hospitals in Vietnam. The most common guidelines used in Vietnam for the treatment of breast cancer are reported in Table 1 (personal communication, Dr. Nguyen Dinh Tung, Oncology Department, Hue Central Hospital).

**Table 1:** Treatment patterns for breast cancer in Vietnam, 2001–2006

Stage of diagnosis	Applied treatment
I	Breast surgery, including mastectomy or breast-conserving surgery with/without axillary dissection. Eligible patients received hormone therapy.
II	Mastectomy breast surgery with axillary dissection. Bilateral oophorectomy by surgery or radiation, if pre-menopause. Adjuvant radiation was supplemented with either external radiotherapy to the breast, chemotherapy, or both. Eligible patients also received hormone therapy.
III	Chemotherapy followed by mastectomy with axillary dissection supplemented with adjuvant chemotherapy. External radiotherapy to the breast was also administered and eligible patients received hormone therapy.
IV	Chemotherapy and/or radiation therapy were supplemented with hormone therapy for eligible patients.

### Initial treatment

The first, or initial, treatment was implemented after the patient received a positive diagnosis for breast cancer. A range of methods was used, depending on the stage of the breast cancer and the characteristics of the patient. The most common treatment methods were surgery, radiation therapy, chemotherapy, and hormone therapy, either alone or in combination. Surgery involved either a complete mastectomy or breast-conserving surgery combined with axillary lymph node dissection. Bilateral oophorectomy might also have been performed. At the time of the study, radiation was delivered with a cobalt-60 unit. Different chemotherapy regimes were used, with the most common being FAC (a combination of cyclophosphamide, doxorubicin, and 5-fluorouracil), FEC 120 (cyclophosphamide, epirubicin, and 5-fluorouracil), and a combination of paclitaxel–doxorubicin. Tamoxifen was often used for hormone therapy. Before treatment began, patients were assessed by laboratory tests based on the proposed approach and regime. Specialists often requested tests, such as hematograms, chest X-rays, kidney or liver function tests (SGOT, SGPT), CA 15.3, and breast and abdominal ultrasound. The choice of tests varied considerably depending on the doctor and the characteristics of each patient. For patients on chemotherapy, the initial treatment often lasted for up to 9 months, but the duration was less for patients not on chemotherapy.

### Follow-up care

Breast cancer treatment has a long course. Normally, patients are required to continue with follow-up care after completing an initial treatment so as to detect local recurrence or metastasis. This type of care was included as ‘follow-up care and supportive treatment’ in this study. During follow-up, outpatient appointments were scheduled every 3 months over the first 2 years and every 6 months in subsequent years. Physical examinations together with laboratory tests, such as hematograms, hepatic ultrasounds, chest X-rays, and CA 15.3, were performed at every out-patient visit. During this time period, most patients were prescribed tamoxifen daily (personal communication, Dr. Nguyen Dinh Tung, Oncology Department, Hue Central Hospital). Since 2006, prescribing tamoxifen has depended on the result of ER tests. In addition, selected patients with signs of recurring tumors received supportive treatment, which might consist of up to six cycles of chemotherapy and/or radiation therapy.

### Cost analysis

Costs were divided into three categories: cost of diagnosis, cost of treatment, and cost of follow-up care. Costs of diagnosis comprised the total cost of laboratory tests that patients received to confirm the diagnosis of breast cancer. Treatment costs included surgery, chemotherapy, radiation therapy, hormone therapy and supportive medication, plus inpatient fees. Cost of initial treatment was specified as the combination of the cost of diagnosis and the cost of treatment, which was calculated on the basis of data collected from medical records of individual patients.

Cost of follow-up care included supportive treatment as well as fees for laboratory tests, out-patient visits, and, in some cases, the cost of a dose of tamoxifen. With our focus only on breast cancer, we assumed that all patients were administered the same tests on every outpatient visit. The compliance of patients with a 5-year period of follow-up care was defined as their conformation to out-patient visits and hormone therapy. A questionnaire that provided information about compliance with the course of treatment on the basis of repeated outpatient appointments and doses of medication was designed. These data were obtained through patient interviews (or interviews with their relatives if the patient was deceased) at the time of the study. We assumed that an affirmative response concerning regular outpatient visits and/or compliance with the tamoxifen regime meant that they were in complete compliance with the standard treatment course (until death or the stated end time). If the respondents said ‘sometimes’ or ‘partial compliance’, we set their care pathway to be 50% of the standard follow-up care. When they said ‘no’, patients were defined as non-compliant, and, accordingly, we set their follow-up care to zero. Costs were estimated based on unit cost of tests, outpatient fees, and price of tamoxifen over time. For supportive treatment, we used the data collected from medical records during the 5 years after diagnosis (if records were available). Costs were discounted at an annual rate of 3% as recommended by the World Health Organization (WHO) (18). These costs were then converted to 2010 figures on the basis of the annual inflation index in Vietnam (19). The cost analysis was performed using the following two methods:

1. Cost analysis by category: For cost categories, aggregate 5-year cost and annual total cost, the mean, standard deviation, the median and cost range were estimated. Values of median for costs were compared with estimates of median regression in the further cost analysis. Costs were presented in US dollars for comparison purposes. The exchange rate used was that in effect on July 15, 2010 (1USD=18544 VND) (20).
2. Cost analysis by key characteristics of patients: Because of non-normally distributed cost data (Shapiro–Wilk test, *p*-values <0.001), a quantile regression model was used to analyze the relationship between characteristics of patients and treatment costs for breast cancer. First, key characteristics of the study population, such as stage at diagnosis, health insurance coverage, and age group, were incorporated in a median regression model to determine factors affecting the 5-year total cost for breast cancer. From this analysis, variables with *p*-value <0.05 were analyzed further in a median regression to estimate the difference in median of cost categories according to their groups. The median difference among groups was presented along with *p* value and their 95% CI. Differences among groups were considered to be statistically significant when the *p* value was ≤0.05.

### Sensitivity analysis

According to statistics from the Ministry of Health, user fees accounted for 60–70% of all hospital revenues in 2006, the rest were from the government budget and other sources (21). Sensitivity analysis, which added 30–40% to unit costs, presented costs for breast cancer treatment with the government budget supplement.

## Results

Study subject characteristics are reported in Table 2. The mean age of patients at the time of diagnosis was 51 years (range 33–75 years). The most frequent age group was that from 40 to 49 years (36.4% of the patients). The study population was evenly divided between urban (51.9%) and rural (48.1%) residences. Slightly more than half of the study population had health insurance (52.7%). At primary diagnosis, the majority of the women had been diagnosed with stage II breast cancer (56.6%). Late-stage diagnosis (stage III and IV) was also common, accounting for 27.1% and 9.3% of the study population, respectively.

**Table 2:** Characteristics of patients with breast cancer in Hue Central Hospital

Characteristics	Number of patients	Percentage
Age at diagnosis (years)		
Mean (SD)	51 (9.5)	
Range	33–75	
<40	16	12.4
40–49	47	36.4
50–59	42	32.6
60–69	17	13.2
≥70	7	5.4
Residence		
Urban	67	51.9
Rural	62	48.1
Health insurance coverage		
Yes	68	52.7
No	61	47.3
Stage of breast cancer at diagnosis		
I	9	7.0
II	73	56.6
III	35	27.1
IV	12	9.3

More patients with health insurance reported complete or partial compliance than did patients without insurance. The proportion of patients dropping out of treatment was larger among patients without health insurance than among those with health insurance (26.2% vs. 5.9%) (Table 3).

**Table 3:** Compliance with breast cancer treatment in relation to health insurance

	Health insurance coverage (%)		
		
Compliance	Yes (95% CI)	No (95% CI)	Total (95% CI)
Complete compliance	76.5 (64.8–85.2)	68.9 (56.1–79.3)	72.9 (64.4–79.9)
Partial compliance	17.6 (10.2–28.7)	4.9 (1.6–14.4)	11.6 (7.1–18.5)
No compliance	5.9 (2.2–14.8)	26.2 (16.6–38.8)	15.5 (10.2–22.9)

95% CI: 95% confidence interval; *p*=0.0016 (Pearson's chi-square test).

**Fig. 1:**
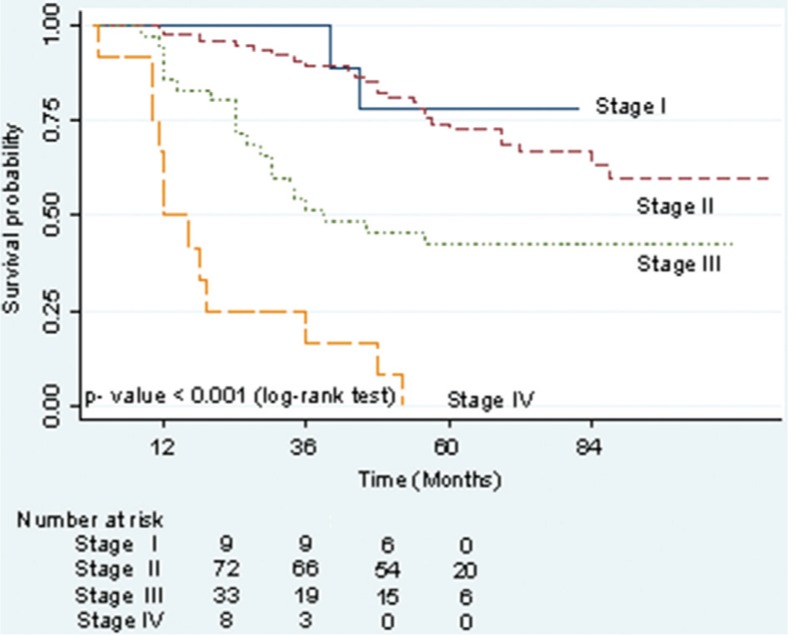
Kaplan–Meier estimates of 5-year survival probability by stage of breast cancer.

Figure 1 presents estimates of survival probabilities of up to 5 years for patients with breast cancer by stage at diagnosis. The survival rate was the lowest for late-stage breast cancer, with 43% of patients at stage III and no cases at stage IV surviving as long as 5 years following diagnosis. Patients at stage I and II at the time of primary diagnosis had higher survival rates after 5 years at 78% and 73%, respectively (log-rank test showed *p*-value <0.001).

Table 4 displays the cost of the different components of treatment, following the primary diagnosis. Women with breast cancer faced a mean cost estimated at $632.85 per patient over the first 9 months of treatment, but the range was very wide ($11.70–$3955.40). The highest average cost incurred was for chemotherapy, at $476.48 per patient. The cost for surgery was also considerable, at $82.35 per patient regardless of whether the method was a complete mastectomy or breast conservation. The lowest treatment cost was for hormone therapy, at only $4.25. Costs for follow-up care over a 5-year period after primary diagnosis included supportive treatment and other follow-up care as described earlier. The mean total cost for follow-up was estimated at $356.24 per patient, with the greatest proportion of costs for follow-up care ($342.18). Aggregated costs over the 5-year treatment course for breast cancer were on average $975.01 per patient but with a wide range ($11.70 to $3955.40). The annual average cost during the 5 years of treatment was an average of $195 per patient.

**Table 4:** Cost estimation per category of breast cancer treatment

	Costs (US dollars)			
	
Category	Mean	SD	Median	Range
Diagnosis	16.02	4.93	16.00	6.9–33.20
Treatment				
Surgery	82.35	31.82	83.00	0.0–235.70
Chemotherapy	476.48	752.81	379.40	0.0–3772.7
Radiation therapy	22.87	37.57	9.00	0.0–300.60
Hormone therapy	4.25	15.20	0.00	0.0–109.60
Other (supportive medication)	4.50	14.34	0.00	0.0–93.10
Inpatient fee	26.38	12.17	24.60	0.0–88.10
Initial treatment cost	632.85	754.54	509.00	11.7–3955.4
Costs for follow-up care				
Supportive treatment	14.06	75.08	0.00	0.0–634.10
Cost for follow-up care	342.18	259.25	409.20	0.0–901.70
Total cost for follow-up care	356.24	260.09	429.40	0.0–901.70
5-Year total cost for treatment course	975.01	730.79	844.20	11.7–3955.4
Annual treatment costs	195.00	146.16	168.80	2.3–791.10

SD, standard deviation; costs are adjusted for inflation to the year 2010; discount rate=3%; exchange rate in July 2010: 1 USD=18,544 VND.

**Fig. 2:**
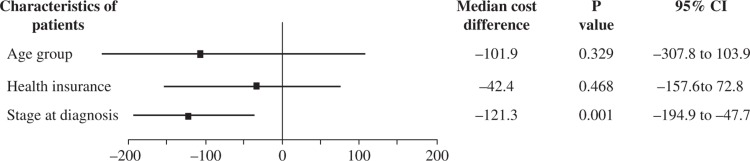
The relationship between key characteristics of patients and the total 5-year cost of the course of treatment for breast cancer.

Figure 2 shows that the stage at diagnosis was significant in terms of the 5-year total cost for breast cancer treatment (*p=*0.001), but there were no significant differences in median total costs related to patient age (*p*=0.329) or whether or not they had health insurance (*p*=0.468).

As shown in Table 5, further analysis of the relationship between stage at diagnosis and different cost categories revealed that costs increased with stage at diagnosis for the initial treatment period. The median costs were $128.70, $368.80, $684.10, and $537.90 for stage I, II, III, and IV, respectively. The difference in initial treatment cost among stages was statistically significant (*p*-values <0.05). By contrast, cost analysis for follow-up care showed that patients with earlier stages at diagnosis faced higher costs because they survived for a longer time period (*p*-values <0.001). The patients with stage II incurred the highest median cost ($516.50), followed by those with stage I ($409.20) for follow-up care. These costs were lower at stage III ($218). Median cost was estimated at $0 for follow-up care in patients at stage IV because 50% of patients at this stage survived less than a year after diagnosis (Figure 1). However, median costs for aggregated 5-year total cost and annual treatment cost revealed that patients at stage II incurred $333.20 and $66.70 higher costs than those with stage I for 5-year total cost or annual treatment cost, respectively (*p*-value=0.009) while variance in treatment costs for late stages (stage III and IV) of breast cancer were not statistically significant from stage I (*p*-values >0.05).

**Table 5:** Variance in cost of breast cancer treatment according to stage at diagnosis

Cost category	Median	Median difference	*P*	95% CI
Initial treatment cost				
Stage I	128.7	0.0	0.218	−76.86–334.26
Stage II	368.8	240.1	0.032	20.92–459.28
Stage III	684.1	519.4	<0.001	286.74–752.06
Stage IV	537.9	482.2	0.001	208.45–334.26
Follow-up cost				
Stage I	409.2	0.0	<0.001	383.91–434.49
Stage II	516.5	107.3	<0.001	80.42–134.18
Stage III	218.4	−109.8	<0.001	−219.43– − 162.17
Stage IV	0.0	−409.2	<0.001	−436.21– − 382.19
Aggregated 5-year total cost				
Stage I	568.6	0.0	<0.001	334.74–802.46
Stage II	901.8	333.2	0.009	83.86–582.54
Stage III	816.1	247.5	0.067	−17.19–512.19
Stage IV	603.4	42.30	0.789	−269.13–353.73
Annual treatment cost				
Stage I	113.7	0.0	<0.001	66.97–160.43
Stage II	180.4	66.7	0.009	16.88–116.52
Stage III	163.2	49.5	0.066	−3.39–102.39
Stage IV	120.7	8.5	0.787	−53.73–70.73

Cost unit: US dollars.

Data related to government subsidy and other sources included in the estimates of treatment cost for breast cancer at public hospitals are presented in Table 6. When these funding sources were included, corresponding mean and median total costs of 5-year treatment and mean and median annual treatment cost were 40% to nearly 70% higher (corresponding to the support of government of 30% and 40%, respectively).

**Table 6:** Estimated costs, including the government subsidy and other sources

	Cost category			
	
	5-Year total cost		Annual treatment cost	
Coverage of government budget and other sources				
	Mean	Median	Mean	Median
30%	1392.9	1206	278.57	241.14
40%	1625.0	1407	325.00	281.33
Baseline	975.01	844.20	195.00	168.80

Cost unit: US dollars.

## Discussion

The results of this study showed that breast cancer was common among young women in central Vietnam during the study period. This is the general profile of breast cancer in developing countries. In developed countries, the majority of breast cancer patients are postmenopausal, 60–70 years old (1, 10). The low coverage of health insurance among the study population was reflective of the study period for Vietnam (22).

The majority of the women in the study population were diagnosed at stage II breast cancer. During the study period, increases in household income due to economic growth and improvements in diagnostic methods for breast cancer (such as the use of ultrasound) provided opportunities for Vietnamese women to contact health facilities and to have their disease detected at an earlier stage than was likely in the past (13).

The costs presented in the study were adjusted to the year 2010 by the growth in the consumer price index and were annually discounted at 3%. The mean total cost of a 5-year course of treatment was estimated at $975.01, with a wide range ($11.70–$3955.40). The compliance with treatment and the type of initial treatment influenced this finding. Some patients refused treatment following diagnosis or did not complete their course of treatment. The majority of those patients that did not complete their treatment course were those not covered by health insurance (Table 3). Establishing a policy of universal health insurance coverage in Vietnam would positively impact the current lack of affordable access to appropriate treatment for chronic diseases such as breast cancer. Nevertheless, the costs determined by this study were much lower than those reported for developed countries. In France, for example, the mean medical cost for a 5-year treatment period for breast cancer was $10,744 (23). Groot et al. estimated the 10-year total cost of treatment per patient with breast cancer based on data retrieved from the WHO-CHOICE database in Africa, Asia, and the Americas in 2000. They reported lower estimates of $602, $356, and $8,530 for Africa, Asia, and North America, respectively (24). These comparisons are similar to a review by Radice et al. in which the cost of breast cancer treatment in developing regions was considered less than or equal to 5% of that in the developed world (3). Comparisons among the wide range of cost estimates for breast cancer treatment and generalizations drawn from economic studies on the disease are made difficult by the different characteristics and patient populations of each country. The diversified unit costs for resource use in different countries could explain the different findings. For instance, in the United Kingdom in 2007, the cost of breast cancer surgery ranged from £1,261 for conservative surgery to £2,073 for a mastectomy, compared to the average cost of $82.35 (about £55 in 2010) for breast cancer surgery that we found in our study (25). In Vietnam, public hospital charges did not measure the full cost of health care resource usage. Unit costs included the price of medications and materials used in the course of treatment but only a portion of those resources that were subsidized by government policy, such as the hospital facility and clinical staff (15, 16). Sensitivity analysis showed that including the government subsidy increased cost estimates by 40 to 70% (Table 6). However, even if user fees and government subsidies were combined, hospital charges were still underestimated. Remuneration of health staff and capital depreciation have not been adequately estimated (26). Unit costs in Vietnamese public hospitals and hospital fees are, therefore, lower than the real cost of the resources used. In a cost analysis of health services in Vietnam, Flessa et al. (2004) determined that the unit cost of an operation such as breast cancer surgery at a central hospital was $175.89, double the cost of our findings (27). In addition, at the time of our study, advanced treatment guidelines were not yet available in Vietnam.

Initial treatment costs were found to be the most expensive component of total costs, accounting for $632.85; these costs represented 65% of the total cost, compared to 63–73.3% in previous studies (6, 7, 23). Chemotherapy costs made up the highest proportion of the initial treatment-attributable costs. In 2005, Oestreicher et al. estimated the cost of chemotherapy for US women with early-stage breast carcinoma to be $23,019, which is 50 times greater than our estimate of $476.48 for Vietnam in 2010 (28). The factors that may contribute to high chemotherapy costs are the types of chemotherapy agents used and the cost of supportive care agents (2). The variety of chemotherapy regimens could explain the wide range of estimated costs for initial treatment as well as the total 5-year treatment course. The regime with paclitaxel–doxorubicin was found to be the most expensive treatment option for chemotherapy over the study period. Many studies have compared the cost-effectiveness of alternative chemotherapy regimes for the treatment of breast cancer. For example, the analysis reported by Mittmann (2010) showed that a protocol with docetaxel offered improved life expectancy but at a higher cost compared with fluorouracin–adriamycin–cyclophosphamid (FAC) (29). According to the experience of the oncologists in HCH (personal communication), the use of expensive chemotherapy regimens depended on the patients’ ability to pay for them. Research on the economic evaluation of different breast cancer chemotherapy regimes should be conducted in the Vietnamese context. The result will help health care providers as well as patients in choosing an affordable and effective treatment method. The high proportion of chemotherapy costs is also a reason as to why initial treatment costs were more for patients diagnosed at stage II and higher; chemotherapy is recommended for most of those patients (see Table 1). In fact, the costs of chemotherapy in our study exceeded the total cost of follow-up care over the 5 years after diagnosis ($476.48 vs. $356.24). Because follow-up treatment for breast cancer in the years after the initial treatment was relatively simple, as described in the method section, the related costs were estimated to be small and relatively stable, as was found in other studies (24, 30). For patients diagnosed with stage I breast cancer, the initial treatment costs were very low, but the follow-up care accounted for a higher proportion of the total cost of treatment. The opposite was true for the patients diagnosed with stage IV. For late-stage breast cancers, the treatment was ineffective. Our study revealed that patients diagnosed at a late stage incurred the same costs as those diagnosed at an early stage but had lower survival times. Early detection of breast cancer may not only increase life expectancy but could also result in resource savings for health care (2, 3). Presently, a pilot screening program for breast cancer has been introduced in some regions of Vietnam. An economic evaluation is necessary before the program will be available nationwide.

Because of underestimation of charges in public hospitals, the annual direct medical cost for breast cancer treatment in this study amounted to about 18% of gross national income (GNI) per capita in Vietnam in 2010 ($195 vs. $1,100) (31). A review by Pisu et al. revealed that out-of-pocket costs for direct medical care were a substantial burden for low-income breast cancer survivors, whose expenses for the disease within 1 year after diagnosis amounted to 75% of their total annual income, compared with only 8% for breast cancer survivors in the highest income group (32). Out-of-pocket costs are the main obstacle to medical treatment, especially in case of diseases with a long natural history (such as breast cancer). Indeed, our study found that a higher number of patients in the group without health insurance coverage dropped out of their treatment regime. Universal health insurance coverage is not yet a reality in Vietnam but should be given more attention, especially since public hospital charges are expected to increase in the near future. The government should have a policy to support cancer patients for whom the cost of illness exceeds their ability to pay or even to co-pay for health insurance. In addition, if a network of primary health care were to be established throughout the country, alternatives such as home care and community care should be promoted to provide health care services to patients who require long-term care, following an initial hospital stay (such as breast cancer patients). The shift to home care settings may improve the compliance with treatment and reduce out-of-pocket costs for patients in Vietnam, where the access to health facilities for cancer treatment has been limited (33).

A number of factors should be considered when interpreting the findings of this study. The data were collected over the period 2001 to 2006 and do not reflect current utilization of advanced treatment methods and new medications for breast cancer treatment. The analysis was limited to costs of primary breast cancer cases, excluding recurrent cases. Both of these factors could lead to an underestimation of the costs. The exclusion of the governmental subsidy and other resources in our cost estimates meant that our estimates did not represent the ‘complete’ resource costs incurred for the treatment of breast cancer in Vietnam, although the estimates do reflect the full costs borne by health care payers. Many changes in the socioeconomic structure of Vietnam occurred during the study period and continue to the present. These changes in the socioeconomic environment might limit the ability to generalize from these study results. In addition, precise data were not available for much of the follow-up period for care; these costs were mainly estimated based on the patient's (or relative's) recall of at least 5 years and therefore are subject to potential bias. Although efforts were made to enroll a suitable number of cases in the analysis, the low incidence rate of breast cancer in Thua Thien Hue province combined with limitations of medical record preservation before 2008 resulted in a small sample size. This affected the opportunity to identify significant differences in cost comparisons among various groups of patients. The estimated costs for breast cancer treatment might not be representative of other main public hospitals in Hanoi and Ho Chi Minh City or of private hospitals in Vietnam, where unit costs may differ from those in our study (15, 16), thereby limiting the ability to generalize our study findings. Despite these limitations, the cost estimates in this article provide the first piece of evidence regarding the cost of breast cancer treatment in Vietnam. These findings reflect the financial burden on health care payers at public hospitals. They will contribute important information to cost-effectiveness analysis of interventions for breast cancer and will help decision-makers engaged in health system planning and resource allocation.

## Conclusion

The direct medical costs of a 5-year course of treatment for primary breast cancer in central Vietnam are much lower than in developed countries. The exclusion of government subsidies and other resources lowered the total costs included in our analysis. However, the long treatment course significantly influenced out-of-pocket payments by patients without health insurance. Having health insurance increased patients’ compliance with treatment because the ability to pay played a major role in treatment uptake. The initial treatment, especially chemotherapy, accounted for the largest part of total costs though the range in costs was wide. There is no significant difference in 5-year total cost with regard to age at diagnosis, health insurance coverage, and between early- and late-stage breast cancer patients in the study. Patients diagnosed with late-stage breast cancer incurred higher costs for initial treatment than those diagnosed at early stages, while their survival time was shorter. Facing these challenges, early detection of breast cancer through screening programs, access to relevant treatment, and an increase in health insurance coverage along with other financial supports to chronic patients should be implemented to improve access to care and the prognosis of breast cancer patients in Vietnam.
